# Relationship between Expression of Cellular Receptor-27.8kDa and Lymphocystis Disease Virus (LCDV) Infection

**DOI:** 10.1371/journal.pone.0127940

**Published:** 2015-05-29

**Authors:** Ronghua Wu, Xiaoqian Tang, Xiuzhen Sheng, Wenbin Zhan

**Affiliations:** Laboratory of Pathology and Immunology of Aquatic Animals, Ocean University of China, 5 Yushan Road, Qingdao 266003, P. R. China; Institute of Oceanology, Chinese Academy of Sciences, CHINA

## Abstract

The 27.8kDa membrane protein from flounder (*Paralichthys olivaceus*) gill (FG) cells was previously identified as a putative cellular receptor involved in lymphocystis disease virus (LCDV) infection. In this paper, the expression of receptor-27.8kDa (27.8R) and LCDV loads in FG cells and hirame natural embryo (HINAE) cells were investigated upon LCDV infection and anti-27.8R monoclonal antibody (MAb) treatment. The results showed the 27.8R was expressed and co-localized with LCDV in both FG and HINAE cell surface. After LCDV infection, the expression of 27.8R exhibited a dose-dependent up-regulation with the increasing of LCDV titers, and demonstrated a tendency to increase firstly and then decrease during a time course up to 9 days; LCDV copies showed a similar variation trend to the 27.8R expression, however, it reached the highest level later than did the 27.8R expression. Additionally, the 27.8R expression and LCDV copies in FG cells were higher than those in HINAE cells. In the presence of increasing concentration of the anti-27.8R MAbs, the up-regulation of 27.8R expression and the copy numbers of LCDV significantly declined post LCDV infection, and the cytopathic effect induced by LCDV in the two cell lines was accordingly reduced, indicating anti-27.8R MAbs pre-incubation could inhibit the up-regulation of 27.8R expression and LCDV infection. These results suggested that LCDV infection could induce up-regulation of 27.8R expression, which in turn increased susceptibility and availability of FG and HINAE cells for LCDV entry, providing important new insights into the LCDV replication cycle and the interaction between this virus and the host cells.

## Introduction

Lymphocystis disease virus (LCDV), which belongs to the genus *lymphocystivirus* within the *Iridoviridae* family [1, 2], is the causative agent of lymphocystis disease characterized by formation of hypertrophied cells on fish skin, fins and mouth, and has affected more than 140 marine and freshwater fish species worldwide, resulting in great economic losses [3, 4]. Recently, the mechanisms of lymphocystis cell formation from the viewpoint of gene expression changes in the infected flounder (*Paralichthys olivaceus*) are investigated [5], and the antiviral molecules, vaccine and immunostimulants that enhance the innate immune response and disease resistance in LCDV infected fish have been reported [6–8]. Despite intense research efforts, no effective antiviral therapy or approved vaccine is yet available, and the pathogenesis is unclear. Understanding of the intimate relationship between the host cells and LCDV will help us clarify the underlying mechanism of virus entry into host cells and devise optimal prophylactic and therapeutic treatment options.

It is well known that viral receptors mediate a physical interaction between virus and target cells and the initial entry. To date, several researches have been focused on the host cellular molecules and viral proteins involved in LCDV infection [9–12]. A 27.8kDa and a 37.6kDa protein from flounder gill (FG) cells [13] have been identified as putative receptors for LCDV by using co-immunoprecipitation and virus overlay protein binding assay (VOPBA), and LCDV infection was partially blocked by antibodies against 27.8kDa or 37.6kDa protein [10, 12, 14]. However, no information is known about the dynamic expression of the receptors in response to LCDV infection.

The FG cells and hirame natural embryo (HINAE) cells have been reported to support LCDV isolation and proliferation [15, 2], which provide available materials for investigating the interaction between the cells and LCDV. In this paper, the changes of 27.8R expression and LCDV loads in the FG and HINAE cells post LCDV infection were investigated, and the effects of anti-27.8R monoclonal antibodies (MAbs) on 27.8R expression and LCDV infection were analyzed. This study would promote better understanding of 27.8R’s functional role in mediating LCDV infection, and the relationship of this virus and the host cells.

## Materials and Methods

### Ethics statement

The present study was conducted in strict accordance with the recommendations in the Guide for the Use of Experimental Animals of Ocean University of China. The protocols for animal care and handling used in this study were approved by the Institutional Animal Care and Use Committee of Ocean University of China (Permit Number: 20111201). Before sacrificing and handling, experimental fish were anesthetized with ethyl 3-aminobenzoate methanesulfonic acid (MS222, Sigma, USA), and all efforts were made to minimize suffering. The NC3Rs ARRIVE Guidelines checklist is presented in the S1 Checklist.

### Cells, virus and antibodies

The FG cells [13] were grown at 22°C in Minimal Essential Medium (MEM, Gibco, Germany) supplemented with 10% fetal bovine serum (FBS, Gibco), 100 IU/ml penicillin and 100 μg/ml streptomycin (Gibco). HINAE cells [16], which were generously donated by Dr. Ikuo Hirono, Tokoyo University of Marine Science and Technology, were cultured at 24°C in Leibovitz's L-15 medium (Gibco), supplemented with 20% FBS, 100 IU/ml penicillin and 100 μg/ml streptomycin. The FBS was reduced to 2% in maintenance medium following viral inoculation onto the cell cultures.

Three flounders suffered from lymphocystis disease, with lymphocystis nodules on the body surface, were bought from a farm in Qingdao, Shandong province of China. LCDV particles were isolated and purified according to the protocol described by Cheng et al. [9]. LCDV was titrated with serial ten-fold dilutions, eight wells per dilution, and the 50% tissue culture infective dose (TCID50) was determined using the Reed-Muench method [17].

Mouse anti-27.8R MAbs (3D9 and 2G11) [14], rabbit anti-LCDV serum [10], and mouse anti-white spot syndrome virus (WSSV) MAb 1D5 [18] were previously produced by our lab. Among them, anti-WSSV MAb 1D5 was used as a negative control.

### SDS-PAGE and Western blotting

The cell membrane proteins of FG and HINAE cells were prepared according to Wang et al. [10]. Breifly, the FG and HINAE cells were harvested and incubated in lysis buffer (250 mM sucrose, 20 mM Tris—HCl, pH 8.0, 137 mM NaCl, 10% glycerol and 1% NP-40) containing the protease inhibitor cocktail (Roche, Germany). After differential centrifugations, membrane proteins were pelleted and suspended in phosphate buffered saline (PBS, pH 7.4). Meanwhile, the cytoplasm proteins were also collected. The protein concentrations were determined by the Bradford method [19] and adjusted to 1mg/ml, then the proteins were subjected to SDS-PAGE in duplicate, one was stained with coomassie blue, the other was transferred onto PVDF membrane (Millipore, USA). The membrane for western blotting was blocked with PBS containing 3% bovine serum albumin (BSA, Sigma) for 1 h at 37°C, followed by incubation with anti-27.8R MAbs (1:1000, 3D9: 2G11 = 1:1, v/v) as the primary antibody, and alkaline phosphatase (AP)-conjugated goat-anti-mouse Ig (Sigma) diluted 1:4000 in PBS as the secondary antibody for 1 h at 37°C. Finally, the bands were stained with freshly prepared substrate solution (100 mM NaCl,100 mM Tris and 5 mM MgCl_2_, pH 9.5) containing nitroblue tetrazolium chloride (NBT, Sigma) and 5-bromo-4-chloro-3-indolylphosphate toluidine salt (BCIP, Sigma) for 5 min, and stopped by washing with distilled water. As a negative control, incubation with anti-WSSV MAb 1D5 (1:1000), instead of the anti-27.8R MAbs, was carried out.

### Co-immunofluorescence staining

The FG and HINAE cells were seeded on the cover slips according to the method described by Morel et al. [20]. Briefly, acid etched circular cover slips were kept in 24-well plates, and 10^4^ cells per well were seeded and incubated to allow cells to attach to the cover slips. After 24 h incubation, the media was removed and the wells were carefully washed with PBS. Then cells were inoculated with 4 TCID_50_/ml LCDV at 22°C for 2 h. After three washes with PBS, cells were fixed with 4% paraformaldehyde (Sangon Biotech, China) at 22°C for 30 min, followed by incubation overnight with anti-27.8R MAbs (1:5000, 3D9: 2G11 = 1:1, v/v) and rabbit anti-LCDV serum (1:500) at 4°C in a moisture chamber. After washing three times with PBS, the cells were incubated for 1 h at 37°C in the dark with fluorescein isothiocyanate (FITC)-conjugated goat-anti-mouse Ig (Sigma) and Cy3-labeled goat-anti-rabbit (Sigma) at a dilution ratio of 1: 256 in PBS in a moisture chamber. 4, 6-diamidino-2-phenylindole (DAPI, Roche) staining (blue) was used to visualize cell nuclei. Slides were rinsed again, and then mounted with 90% glycerin and observed under a fluorescence microscope.

### LCDV infection and sampling

To determine the effect of LCDV titers on expression of 27.8R, FG and HINAE cells were inoculated with LCDV at a multiplicity of infection (MOI) of 0.003, 0.03, 0.3 and 3.0 respectively. Triplicate wells were sampled at 48 h post infection (p.i.), and the membrane proteins were extracted as described by Wang et al. [10] for detecting the expression of 27.8R by a sandwich indirect enzyme-linked immunosorbent assay (ELISA). As negative controls, an equal number of un-infected cells grown in maintenance medium were sampled in parallel.

The relationship between 27.8R expression and LCDV replication was investigated. Approximately 1.5 ×10^6^ FG or HINAE cells per well were seeded in 6-well plates and incubated overnight. The cells were infected with LCDV at a MOI of 3.0. Triplicate wells were sampled at 2, 6, 12, 24, 36, 48, 72, 120, 168 and 216 h p.i. After the culture medium was removed, the cells of each well were collected by scraping, and cell membrane protein was extracted as described above for detecting the expression of 27.8R by sandwich ELISA. As a negative control, an equal number of un-infected cells grown in maintenance medium were sampled in parallel. Meanwhile, total cell DNA was extracted using TIANamp Marine Animals DNA Kit (Qiagen, Germany) following the manufacturer’s protocols. The DNA purity and quantity were assessed using a Nanodrop 8000 spectrophotometer (Thermo Scientific, Wilmington, USA). The quantification of the propagated LCDV in the cells was accomplished through real-time quantitative PCR (qPCR).

### Real-time quantitative PCR

To investigate changes of LCDV copy number after infection, a real-time quantitative PCR assay was developed according to Zhu et al. [21] with some modifications, and the absolute copy number of LCDV major capsid protein (MCP) gene was determined based on the standard curve method. Briefly, DNA fragment amplified by P1 and P2 primers (P1: 5'-CAT CAT GCC TTT GAC AGC-3'; P2: 5'-GGA TCA GCA GCA ATA CCC-3', 348bp) [22] targeting MCP gene of LCDV was used for constructing positive plasmid DNA for preparing standards. Then positive control plasmid containing MCP gene of LCDV was constructed, purified and sequenced to ensure the presence of the target sequence. The concentration of the positive plasmid DNA was determined with spectrophotometric analysis, and ten-fold dilution series from 1.9 ×10^1^ to 1.9 × 10^9^ copies were prepared as standards.

The prime sets, forward primer P3 (5'-TCC ACC GTC AAA GAT TAC-3') and reverse primer P4 (5'-CAA TTC CAC CGT CAA AGC-3') targeting partial MCP gene (173 bp) [22], was designed for LCDV MCP qPCR. The qPCR reaction contained 50 ng template DNA, 10 μl SYBR Green Premix (TaKaRa, Japan), 200 nM of each primer and sterile distilled water to a final volume of 20 μl, and each sample was run in four replicates. The PCR reaction was performed using following conditions: 7 min incubation at 95°C, followed by 45 cycles at 95°C for 10 s, 53°C for 10 s and 72°C for 20 s. Melting curve analysis was carried out after each PCR run to ensure the specificity of the reaction.

### Sandwich ELISA

The concentration of FG and HINAE cell membrane proteins were adjusted to 50 μg/ml in PBS. A sandwich ELISA to detect 27.8R expression was carried out according to Charlermroj et al. [23] with minor modifications. Anti-27.8R MAb 3D9 was utilized as a capture antibody, and anti-27.8R MAb 2G11, which was utilized as a detection antibody, was conjugated with a alkaline phosphatase (AP) using a Lightning-Link Alkaline Phosphatase Conjugation Kit (Innova Biosciences, UK) according to manufacturers’ instructions. The wells of flat bottom microplates (Costar, USA) were coated overnight at 4°C with 100 μl/well of the capture antibody (1:1000) diluted in 50 mM sodium carbonate-bicarbonate buffer (pH 9.6). After three washes with PBS, each well was blocked by addition of 200 μl of 3% BSA in PBS for 1 h at 37°C. The same washing steps were repeated afterwards before adding and incubating 100 μl of cell membrane proteins in triplicate for 1 h at 37°C. The plate was washed and then AP-labeled MAb 2G11 (1:1000) diluted in PBS was added and incubated for 1 h at 37°C. After washing, 100 μl of 0.10% (w/v) *p*-nitrophenyl phosphate (pNPP, Sigma) in carbonate-bicarbonate buffer containing 0.5 mM MgCl_2_ was added to each well and incubated for 30 min at room temperature in the dark. The reaction was stopped with 50 μl per well of 2 M NaOH and absorbance values were measured at 405 nm with an automatic ELISA reader (TECAN, Switzerland).

### Blocking assay against 27.8R expression and LCDV infection

The blocking assay using anti-27.8R MAbs was performed by the following methods described by Tian et al. [24] with minor modifications. FG and HINAE cells grown in 6-well plate were incubated with serially increasing concentration (0, 0.16, 1.6, 16 and 160 μg/ml) of anti-27.8R MAbs for 3 h at 22°C. After washing twice with PBS, cells were challenged with LCDV at a MOI of 3.0, triplicate wells were sampled at 48 h p.i., and the membrane proteins were extracted for detecting the expression of 27.8R using sandwich ELISA. As control group, an equal number of un-infected cells, which were pre-incubated with anti-27.8R MAbs and grown in maintenance medium, were sampled in parallel. Meanwhile, the total DNA of experimental groups was extracted for quantification of the propagated LCDV by qPCR, the LCDV-infected cells without pre-incubation with anti-27.8R MAbs served as positive control group.

For the LCDV infection inhibition assay *in vitro*, FG and HINAE cells were grown in 24 well plate at 22°C until the cell number reached about 10^5^ per well. After the medium was removed, the cells were washed gently with PBS, and incubated with increasing concentration (0.04, 0.4, 4 and 40 μg/ml) of anti-27.8R MAbs for 3 h at 22°C. Following incubation, the wells were washed twice with PBS, and the plates were inoculated with LCDV at 4 TCID_50_/ml and incubated at 22°C for 3 h. After virus adsorption, the dissociative viral particles were removed by washing three times with PBS and maintenance medium was added, then the cytopathic effect (CPE) in FG and HINAE cells were monitored by phase contrast microscope. Anti-WSSV MAb 1D5 at 40 μg/ml instead of anti-27.8R MAbs served as positive control. Each group was performed in triplicate.

### Statistic

All data were expressed as mean ± standard deviation. The statistical analysis was performed by one-way ANOVO and Student’s *t* test using SPSS 17.0 software. Differences were considered significant when *p* < 0.05.

## Results

### Expression of 27.8R in FG and HINAE cells

**Fig 1:**
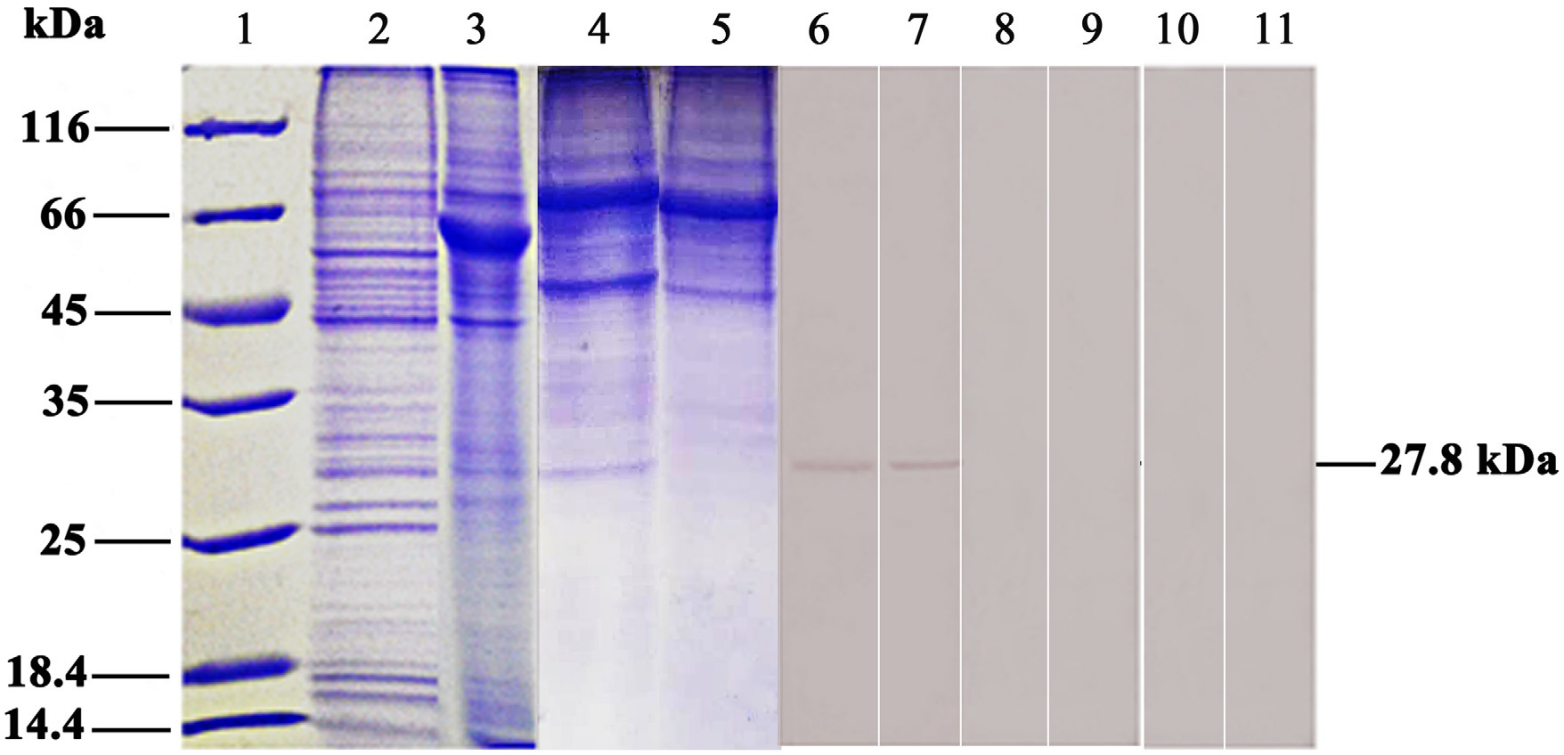
SDS-PAGE and western blotting of the LCDV cellular receptor-27.8kDa protein. Lane 1: Molecular mass marker; Lane 2, 3: SDS-PAGE of FG and HINAE cell membrane protein, stained with coomassie bule; Lane 4, 5: SDS-PAGE of FG and HINAE cell cytoplasm protein, stained with coomassie bule; Lane 6, 7: reaction with anti-27.8R MAbs showed only one 27.8 kDa in FG and HINAE cell membrane protein; Lane 8, 9: reaction with anti-27.8R MAbs showed no band in FG and HINAE cell cytoplasm protein; Lane 10, 11: anti-WSSV MAb 1D5 instead of anti-27.8R MAbs served as negative controls.

To confirm the expression of 27.8R protein in both FG and HINAE cells, SDS-PAGE followed by western blotting was performed using the cell membrane proteins and cytoplasm proteins extracted from the two cell lines. The results showed that anti-27.8R MAbs reacted with only one band at a molecular weight of 27.8kDa from both FG and HINAE cell membrane proteins (Fig 1, lane 6 and 7), suggesting 27.8R was expressed in the cell membrane. No bands appeared in the cytoplasm proteins (Fig 1, lane 8 and 9) and negative controls.

### Co-localization of LCDV and 27.8R protein in FG and HINAE cells

**Fig 2:**
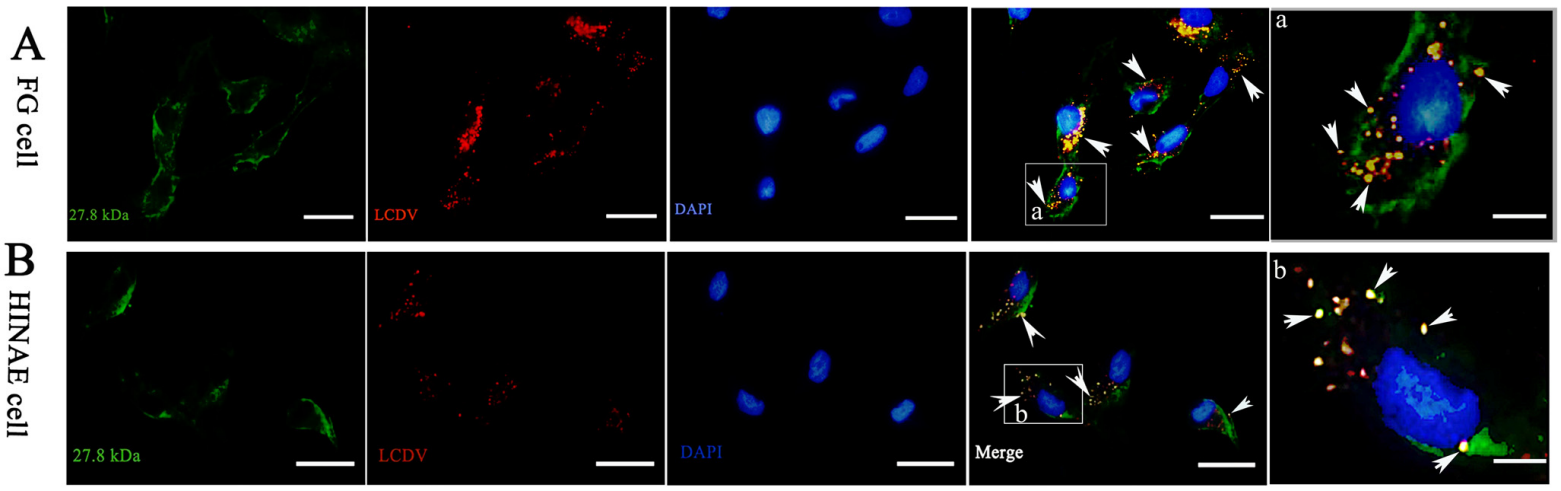
Co-localization of LCDV and 27.8R in FG and HINAE cell surface. FG cells (A) and HINAE cells (B) were exposed to LCDV at 22°C for 2 h and stained with mouse anti-27.8R MAbs and rabbit anti-LCDV serum for detection of 27.8R (green) and LCDV (red) simultaneously. The merged yellow signals (arrows) indicated the co-localization of LCDV and 27.8R protein on cell surface. Cell nuclei were counterstained in blue by DAPI. Scale bar = 20 μm. (a) and (b) were the higher magnification view of the co-localized area in FG and HINAE cells, respectively, scale bar = 5 μm.

The immunofluorescence staining using mouse anti-27.8R MAbs showed that the specific green fluorescence signals were mainly clustered at the membrane of FG and HINAE cells, indicating the distribution of 27.8R; the staining by using rabbit anti-LCDV serum showed that the specific red fluorescence signals were located on both the cell membrane and in the cytoplasm. The merged yellow signals revealed co-localization of LCDV and 27.8R on the cell surface, while the red signals in the merged figure indicated LCDV particles in the cytoplasm (Fig 2). Cell nuclei were counterstained in blue by DAPI.

### Effect of LCDV titers on 27.8R expression

**Fig 3:**
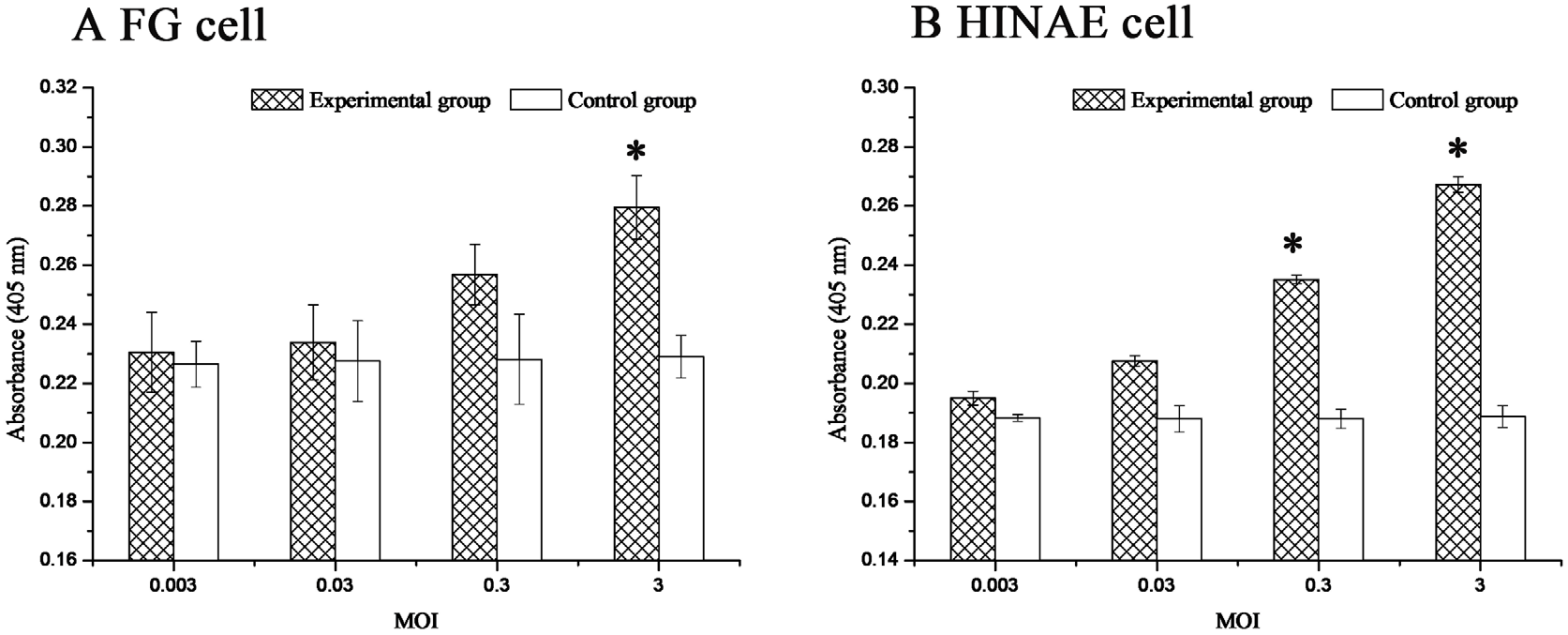
Dose response of LCDV-induced 27.8R expression in FG and HIANE cells. FG and HINAE Cells were infected with LCDV at a MOI of 0.003, 0.03, 0.3 and 3.0 respectively. The cells without LCDV infection served as control. The cells were sampled at 48 h post infection. Error bars represented standard deviation (SD). Data represented the absorbance value at 405 nm (mean ± SD; n = 3) and were compared by Student’s *t* test. The asterisk represented the statistical significance (*p* < 0.05) as compared with the control.

Dose-response studies in FG and HIANE cells exposed to LCDV at a MOI of 0.003, 0.03, 0.3 and 3.0 were carried out and 27.8R expression was detected at 48 h p.i. The sandwich ELISA results showed that the 27.8R expression was up-regulated upon LCDV infection in the two cell lines and a dose-dependent increase was observed with increasing of LCDV titer (Fig 3), which confirmed that the markedly increased 27.8R expression in the two cell lines were associated with LCDV infection. The enhanced expression of 27.8R was observed at 0.003 MOI in the two cell lines, and LCDV infection induced a significant up-regulation of 27.8R expression at a MOI of 3.0 in FG cells (Fig 3A) and at MOI of 0.3 and 3.0 in HIANE cells (Fig 3B) (*p* < 0.05).

### Time-course changes of LCDV copy numbers after inoculation

**Fig 4:**
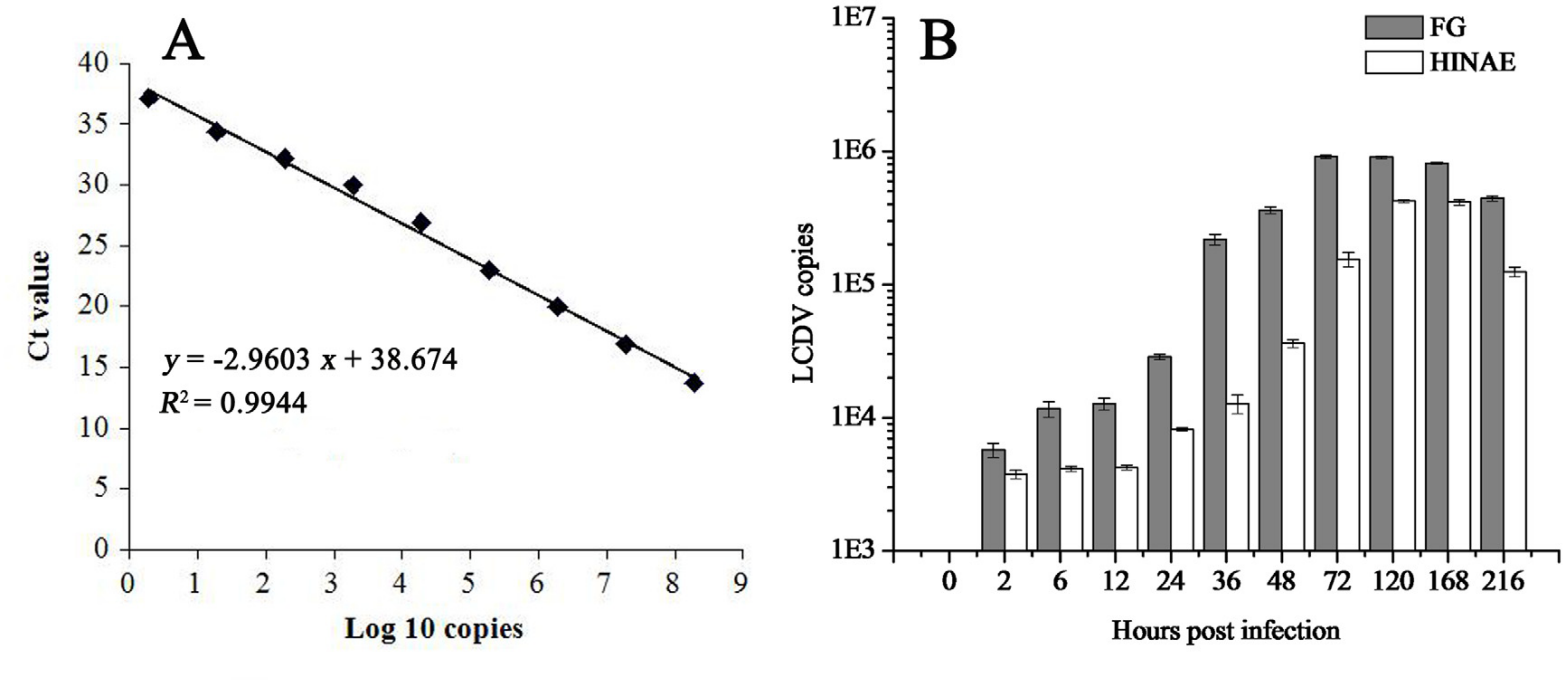
Dynamics of LCDV copies in FG and HINAE cells post LCDV infection investigated by qPCR. (A) Standard curve of LCDV MCP qPCR assays. The X-axis showed the positive control plasmid copy number in Log 10 value, and the Y-axis indicated the corresponding cycle threshold (Ct) value. R^2^: coefficient of determination. (B) Changes of LCDV copies in FG and HINAE cells post LCDV infection. 0 h represented un-infected cells. Error bars represented SD. Data represented the number of LCDV copies per microgram of total DNA in the cell samples (mean ± SD; n = 3).

Serial ten-fold dilutions of the positive control plasmids were run with the LCDV MCP qPCR assays and standard curve was generated based on the Ct values (Fig 4A). The linear range for the MCP qPCR assay was between 1.9×10^1^ and 1.9×10^9^ copies, and the coefficient of determination (R^2^) for the standard curve was 0.994.

The number of LCDV copies in FG and HINAE cells was calculated according to the standard curve generated from samples of positive plasmid (Fig 4B). The results showed LCDV loads increased slowly in FG cells by 12 h (< 3×10^4^ copies), and increased notably and peaked at about 9×10^5^ copies at 72 h (*p* < 0.05), then an obvious decrease occurred at 168 h when the cells almost disintegrated. While in HINAE cells the LCDV quantity reached about 3.5×10^4^ copies at 36 h, and then significantly increased from 48 h and rose to a peak of about 4.2×10^5^ copies at 120 h (*p* < 0.05), followed by a significant decline at 216 h (about 1.2×10^5^ copies). The detected quantities of LCDV in FG cells were significantly higher than those in HINAE cells from 2 h to 216 h (*p* < 0.05).

### Dynamic expression of 27.8R during LCDV replication

**Fig 5:**
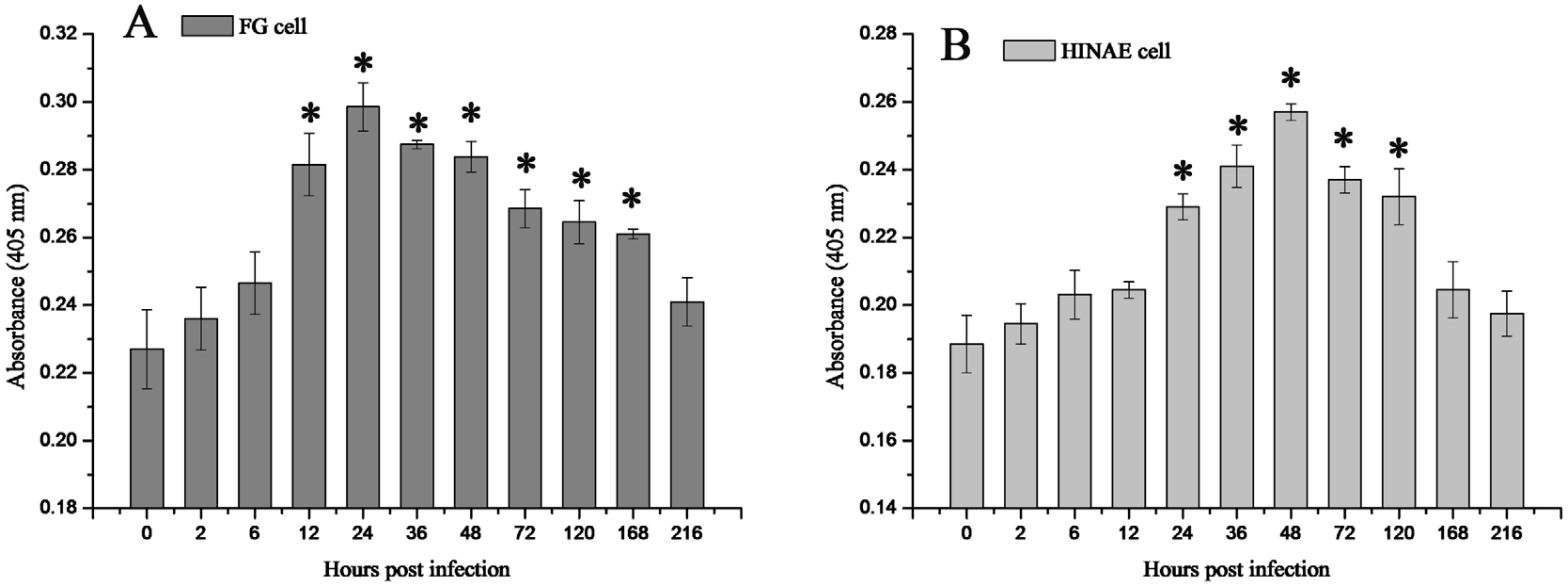
Dynamic expression of 27.8R in FG (A) and HINAE (B) cells post LCDV infection detected by sandwich ELISA. The cells were infected with LCDV at a MOI of 3.0 and sampled at different time points post infection. Error bars represented SD. Data represented the absorbance value at 405 nm (mean ± SD; n = 3) and were compared by Student’s *t* test. Un-infected cells (0 h) represented the negative control. The asterisk represented the statistical significance (*p* < 0.05) as compared with the negative control.

The concentration of 27.8R in FG and HINAE cell membrane proteins post LCDV infection was determined by sandwich ELISA as absorbance value at 405 nm. The results showed 27.8R expression in the two cell lines was up-regulated after LCDV infection in a time-dependent manner (Fig 5). In FG cells, up-regulation of 27.8R expression was significant from 12 to 168 h (*p* < 0.05) (Fig 5A), and the concentration of 27.8R peaked at 24 h (*p* < 0.05) and then slowly descended to near the level of negative control by 216 h when the cells almost disintegrated. In HINAE cells, similar variation tendency was found, however, the 27.8R expression was significantly up-regulated from 24 to 120 h (*p* < 0.05) (Fig 5B), and reached the peak at 48 h (*p* < 0.05) with a lower value than that of FG cells, and then slowly decreased to near the level of control group by 168 h (*p* > 0.05).

### Blocking effect of anti-27.8R MAbs on 27.8R expression and LCDV infection

**Fig 6:**
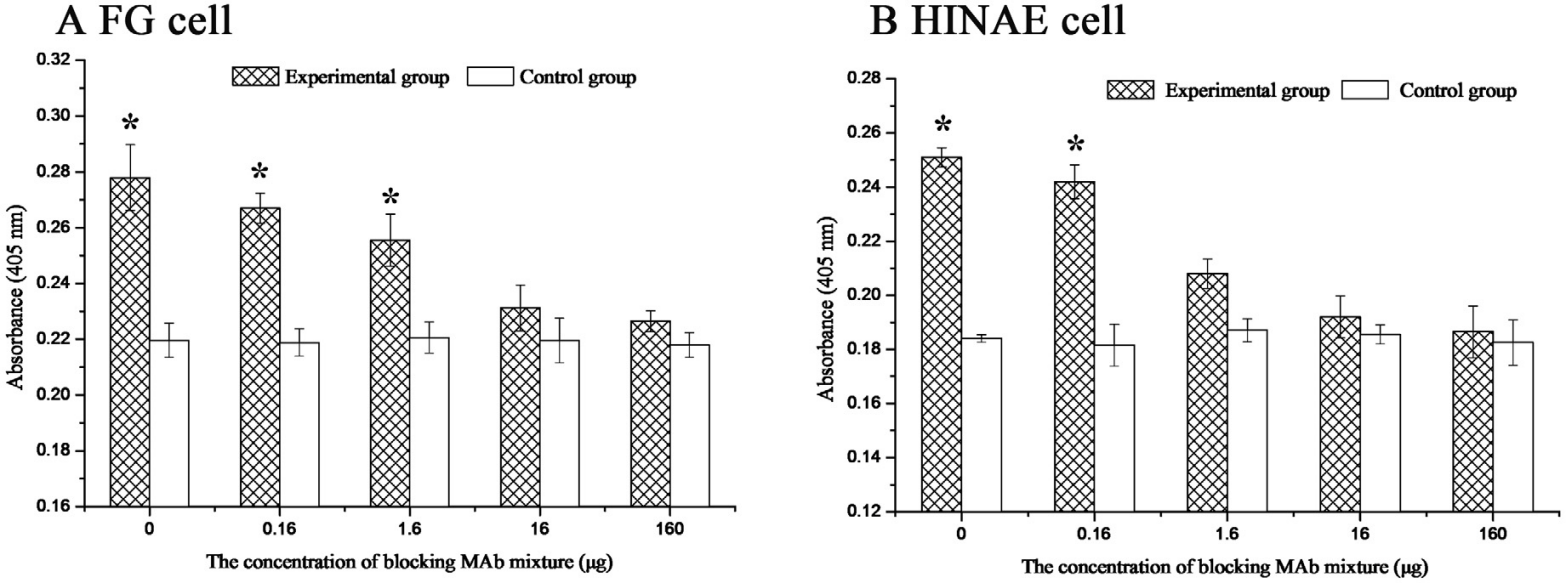
Blocking effect of anti-27.8R MAbs on 27.8R expression. The cells were pre-incubated with different concentration of anti-27.8R MAbs. Experimental groups were challenged by LCDV at a MOI of 3.0, and the cells without LCDV infection served as control groups. The cells were sampled at 48 h post infection. Error bars represented SD. Data represented the absorbance value at 405 nm (mean ± SD; n = 3) and were compared by Student’s *t* test. The asterisk represented the statistical significance (*p* < 0.05) as compared with the control group.

When the two cell lines were pre-incubated with increasing concentration of anti-27.8R MAbs and infected with LCDV, the up-regulation of 27.8R expression showed a dose-dependent decrease at 48 h p.i. (Fig 6). In the presence of 0.16 and 1.6 μg MAbs, the 27.8R expression in FG cells was lower than the un-blocked cells (0 μg MAbs) but still significantly higher than the un-infected control group (*p* < 0.05); however, in the presence of 16 and 160 μg MAbs, it was dramatically reduced to near the level of the un-infected control group, showing no significant difference between them (*p* > 0.05) (Fig 6A), which suggested that 27.8R expression was obviously blocked. In HINAE cells, the 27.8R expression in the presence of 0.16 μg MAbs was lower than the un-blocked cells (0 μg MAbs) but significantly higher than the un-infected control cells (*p* < 0.05); in the presence of 1.6, 16 and 160 μg of MAbs, no significant differences in 27.8R expression were found between the pre-treated groups and the un-infected control cells (*p* > 0.05) (Fig 6B).

**Fig 7:**
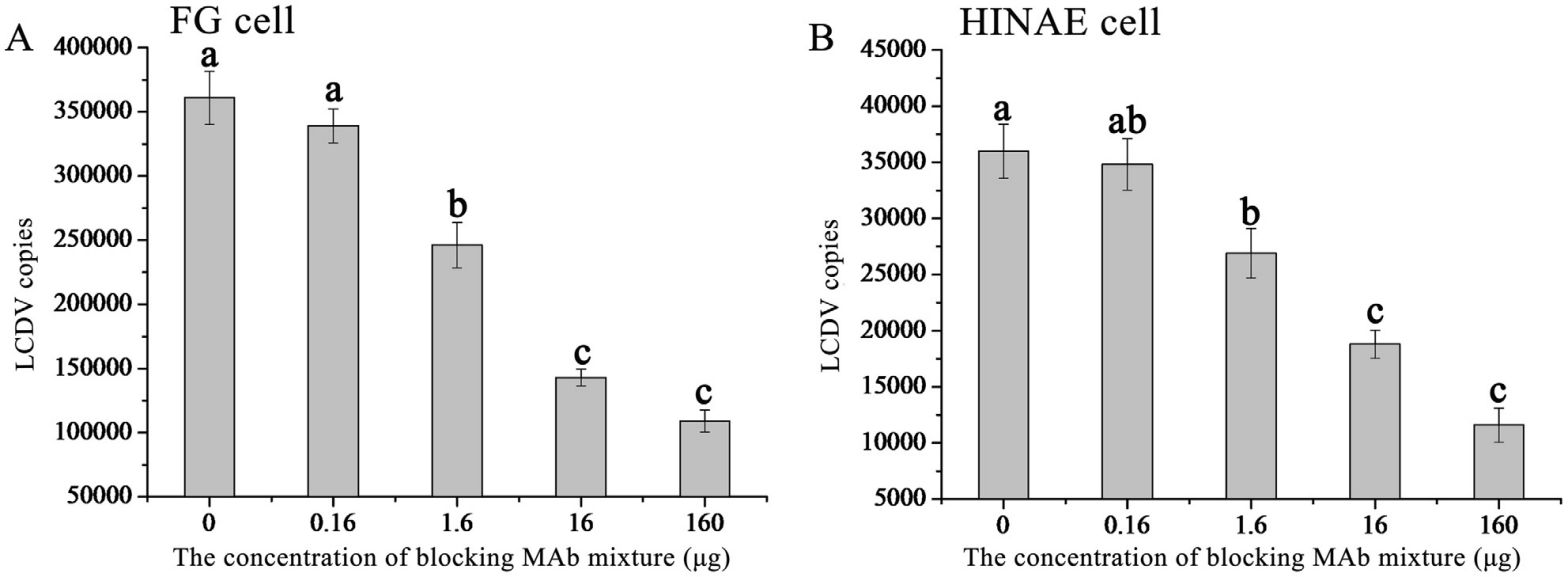
Inhibition of anti-27.8R MAbs on LCDV replication. At 48 h post infection, the quantity of LCDV loads in FG (A) and HINAE (B) cells pre-incubated with different concentration of anti-27.8R MAbs was showed. Error bars represented SD. Data represented the number of LCDV copies per microgram of total DNA in the cell samples (mean ± SD; n = 3) and were compared by One-way ANOVO. Means with different letters were significantly different (*p* < 0.05).

Meanwhile, LCDV load in the two cell lines was measured by qPCR, and it was found that LCDV load gradually decreased with the increase of the concentration of anti-27.8R MAbs (Fig 7). In the presence of 0.16 μg of MAbs, the decrease of LCDV load (3.39 × 10^5^ copies) in FG cells was not evident (*p* > 0.05) as compared with the un-blocked positive controls (3.61 × 10^5^ copies); while in the presence of 1.6 μg of MAbs, LCDV load (2.46 × 10^5^ copies) showed a significant decline (*p* < 0.05); LCDV loads continuously decreased in the presence of 16 μg of MAbs and showed no significant changes in the presence of 160 μg of MAbs (Fig 7A). Similarly, in HINAE cells, LCDV load was significantly reduced in the presence of more than 1.6 μg of MAbs as compared with the un-blocked positive control (3.6 × 10^4^ copies) (*p* < 0.05); however, there were no significant differences in LCDV loads in the presence of 0.16 μg (3.48 × 10^4^ copies) and 1.6 μg (2.7 × 10^4^ copies) of MAbs (*p* > 0.05) (Fig 7B).

**Fig 8:**
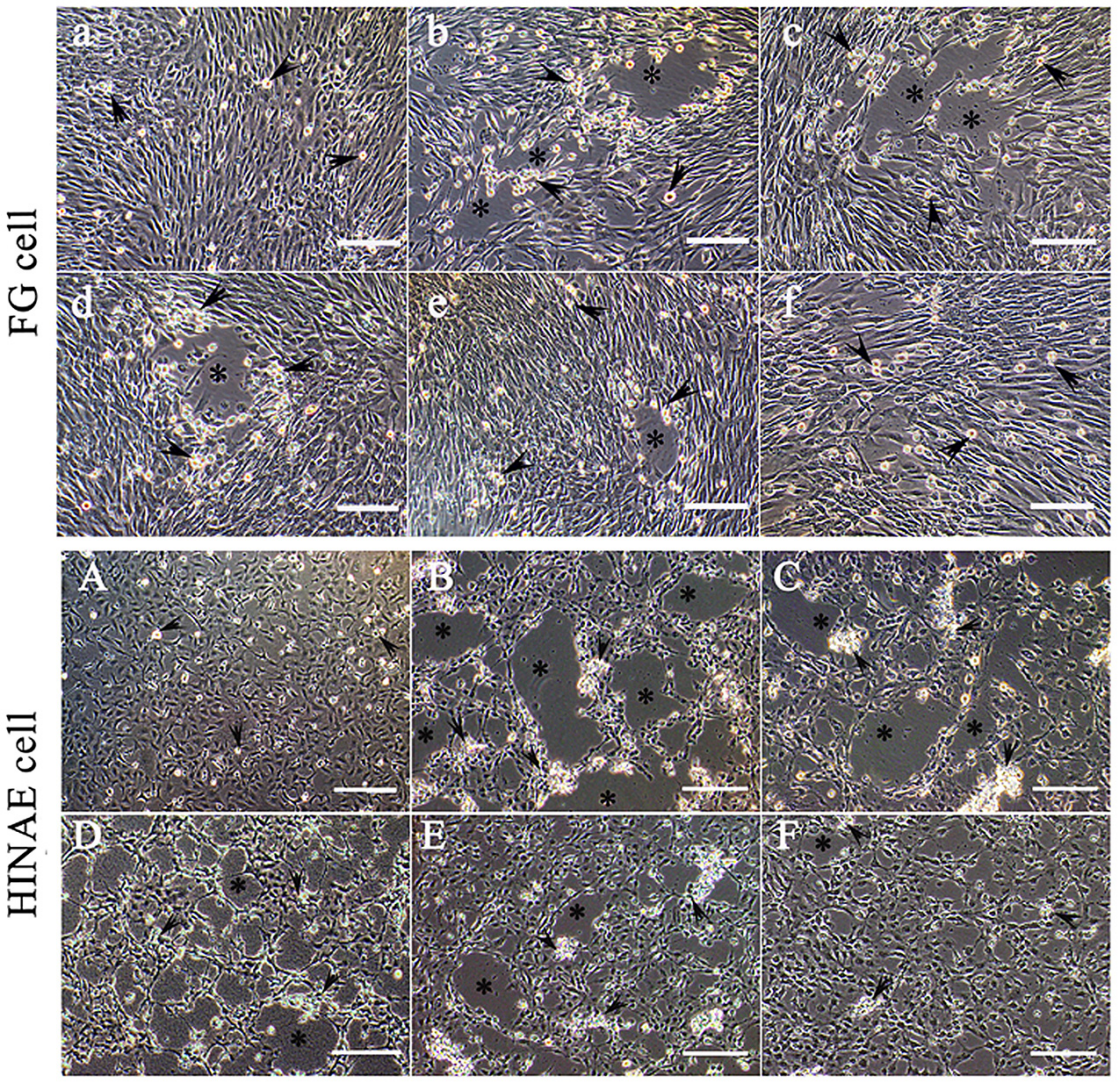
LCDV infection inhibition assays using anti-27.8R MAbs showing the cytopathic effect (CPE) in FG cells at 120 h p.i. (a-f) and HINAE cells at 168 h p.i. (A-F). Black arrows: dying cells with typical cell detachment and rounding; (*) CPE. (a) FG cell cultures without LCDV infection. (b) ++++: Pre-incubation with anti-WSSV MAb 1D5. (c−f) FG cells pre-incubated with 0.04, 0.4, 4 and 40 μg of anti-27.8R MAbs, respectively. (c) ++++, (d) ++, (e)++ (f) +. Scale bars = 100 μm. (A) HINAE cell cultures without LCDV infection. (B) ++++: Pre-incubation with anti-WSSV MAb 1D5. (C−F) HINAE cells pre-incubated with 0.04, 0.4, 4 and 40 μg of anti-27.8R MAbs, respectively. (C) ++++, (D) +++, (E) ++, (F) +. Scale bars = 200 μm.

*In vitro*, anti-27.8R MAbs exhibited a dose-dependent inhibitory effect on LCDV infection in the two cell lines (Fig 8), and the CPE in cells was shown in Table 1. For FG cells at 120 h p.i., no CPE was observed in the FG cultures without LCDV infection (Fig 8a); however CPE characterized by many large viral plaques was clearly developed in the un-blocked positive controls (Fig 8b); in the presence of 0.04 μg per well of anti-27.8R MAbs, CPE was similar to positive controls (Fig 8c); in the presence of 0.4 μg and 4 μg MAbs, CPE was inhibited and the number of viral plaques was reduced significantly (Fig 8d and 8e); in the presence of 40 μg MAbs, little CPE was present, demonstrating cellular retraction, aggregation and loss of adherence to the substrate (Fig 8f). Similar inhibition effects were obtained in HINAE cells, and the level of LCDV infection was reduced with the increasing concentration of anti-27.8R MAbs at 168 h p.i. (Fig 8A–8F).

**Table 1:** *In vitro* LCDV infection inhibition assays using anti-27.8R MAbs.

MAb type	MAb concentration (μg well^-1^)	CPE in FG cells at 5 d p.i	CPE in HINAE cells at 7 d p.i
Anti-27.8R MAbs	0.04	+ + + +	+ + + +
	0.4	+ +	+ + +
	4	+ +	+ +
	40	+	+
Anti-WSSV MAb	40	+ + + +	+ + + +

Anti-WSSV MAb served as a positive control MAb.

++++: 75–100% of cells showing CPE;

+++: 50–75% of cells showing CPE;

++: 25–50% of cells showing CPE;

+: few dying cells.

## Discussion

The ability of a virus to replicate in particular cells depends on interactions between virus and cellular factors at each step of the viral cycle, from the initial entry to the ultimate release and transmission of virions. Our previous researches demonstrated that the 27.8 kDa protein from FG cells, which had a strong association with β-actin and might be a part of the β-actin protein or an unknown protein sharing some epitopes with β-actin protein, was a putative cellular receptor specific for LCDV binding and infection [10]. 27.8R was proved to express on the membrane of FG cell by confocal fluorescence microscopy and immunogold electron microscopy [14]. Virus binding assay showed LCDV could attach to the surface of FG cells [12], and cause CPE in FG cells post infection [14]. In present study, 27.8R was detected in both FG and HINAE cells and co-localized with LCDV on the cell surface; during 2 h of LCDV inoculation, a few of LCDV particles entered the cytoplasm, revealing 27.8R could bind and mediate LCDV entry into the cells, and the two cell lines should be appropriate materials to study the relationship between 27.8R expression and LCDV replication.

In response to LCDV infection, the expression of 27.8R showed a dose-dependent increase that came along with an increase of LCDV titers, indicating that the 27.8R could be up-regulated by LCDV exposure and LCDV entry into the cells activated the 27.8R expression. In addition, the 27.8R expression reached the peak level early before the highest LCDV copies occurred in the two cell lines, which suggested that the up-regulation of 27.8R induced by LCDV might facilitate LCDV entry. Meanwhile, the LCDV copies and 27.8R expression were higher in FG cells than in HINAE cells, revealing that FG cells were more susceptible to LCDV than HIANE cells, suggesting a higher expression of 27.8R in FG cells might result in a higher level of LCDV entry and production. These findings were also supported by some other receptor-dependent virus infection, showing that viral infection could induce an up-regulation of cellular receptor expression, this change, in turn, enhanced susceptibility and availability of the host cells for viral entry [25–29]. This might constitute a strategy for the virus to increase its infectivity, so the mechanism through which LCDV is capable of up-regulating the receptor expression needs more researches. Additionally the 27.8R expression up-regulation might differently affect the LCDV-infected cells leading to various functional alterations, this is also a potential area for future research.

It has been found that lowering the level of receptor expression was expected to render the hosts rather resistant to receptor-dependent viral infections [30], and inhibition of receptor expression via anti-receptor antibodies or RNA interference could decrease virus infection [31, 32]. In the present study, 27.8R expression available for LCDV binding was inhibited by pre-incubation with anti-27.8R MAbs, leading to a significant reduction of LCDV load in FG and HINAE cells. Similar blocking effects that inhibited LCDV-induced CPE were also observed *in vitro*. These results revealed that anti-27.8R MAbs pre-incubation could block the initial interaction between LCDV and its receptor, thus providing protection from LCDV infection. Since lowering 27.8R expression could decrease LCDV infection, it is worthy of developing RNA interference to inhibit 27.8R expression in further researches.

On the other hand, in anti-27.8R MAbs blocking assay, although pre-incubation of the cells with increasing concentration of anti-27.8R MAbs significantly decreased the CPE in FG and HINAE cells, little CPE was still present and a low level of LCDV load was detected, even the cells were treated with anti-27.8R MAbs at the saturation concentration which was deduced from our previous paper [14]. These results revealed that the blocking effect was efficient but not complete, so LCDV infection might involve other receptors. The 37.6 kDa LCDV-binding protein, previously identified from FG cells by using VOPBA [12], might contribute to the presence of the CPE and LCDV load. Therefore, more work concerning the relationship between 37.6 kDa protein expression and LCDV infection is needed.

In conclusion, this paper investigated the dynamics of 27.8R expression and LCDV replication in FG and HINAE cells, and the relationship between 27.8R expression and LCDV infection. 27.8R was proved as a LCDV-binding molecule shared by this two cell lines and could mediate LCDV entry, and its expression was up-regulated post LCDV infection, high expression of 27.8R had a positive correlation with LCDV propagation. Pre-incubation of anti-27.8R MAbs could decrease LCDV infection, although incomplete inhibition of viral production has been observed *in vitro*, anti-27.8R MAbs might serve as potential antiviral agents. These results suggested that LCDV infection could induce up-regulation of 27.8R expression, which in turn enhanced the susceptibility and availability of FG and HINAE cells for LCDV entry, providing new important insight into the LCDV replication cycle and the interaction between this virus and the host cells. To our knowledge, this is the first study which revealed the up-regulation of virus cellular receptor in LCDV infection. Future studies on the trigger factors up-regulating the 27.8R expression post LCDV infection will shed light on the underlying mechanism.

## Supporting Information

S1 Checklist NC3Rs ARRIVE Guidelines Checklist. The ARRIVE guidelines describes the number and specific characteristics of animals used; details of housing and husbandry; and the experimental, statistical, and analytical methods.
(PDF)
